# Personalized Medicine in the U.S. and Germany: Awareness, Acceptance, Use and Preconditions for the Wide Implementation into the Medical Standard

**DOI:** 10.3390/jpm6020015

**Published:** 2016-05-02

**Authors:** Kateryna Kichko, Paul Marschall, Steffen Flessa

**Affiliations:** Faculty of Law and Economics, Department of Health Care Management, Ernst-Moritz-Arndt-University, Greifswald 17489, Germany; paul.marschall@uni-greifswald.de (P.M.); steffen.flessa@uni-greifswald.de (S.F.)

**Keywords:** personalized medicine, personalized drug, pharmacogenetic test

## Abstract

The aim of our research was to collect comprehensive data about the public and physician awareness, acceptance and use of Personalized Medicine (PM), as well as their opinions on PM reimbursement and genetic privacy protection in the U.S. and Germany. In order to give a better overview, we compared our survey results with the results from other studies and discussed Personalized Medicine preconditions for its wide implementation into the medical standard. For the data collection, using the same methodology, we performed several surveys in Pennsylvania (U.S.) and Bavaria (Germany). Physicians were contacted via letter, while public representatives in person. Survey results, analyzed by means of descriptive and non-parametric statistic methods, have shown that awareness, acceptance, use and opinions on PM aspects in Pennsylvania and Bavaria were not significantly different. In both states there were strong concerns about genetic privacy protection and no support of one genetic database. The costs for Personalized Medicine were expected to be covered by health insurances and governmental funds. Summarizing, we came to the conclusion that for PM wide implementation there will be need to adjust the healthcare reimbursement system, as well as adopt new laws which protect against genetic misuse and simultaneously enable voluntary data provision.

## 1. Introduction

The successful completion of the Human Genome Project (HGP) in 2003 [1] and the fast decreasing human genome sequencing costs [2] encouraged the development of a new medical approach which is called Personalized Medicine (PM). Though the term has been in use for about a decade, depending on the scope, its definition can vary widely. The Personalized Medicine Coalition defines Personalized Medicine as “the use of new methods of molecular analysis to better manage a patient’s disease or predisposition to disease’’ [3]. The FDA sees it as tailoring medical treatment to the patient’s individual genetic, anatomical and physiological characteristics [4], which means “providing the right patient with the right drug at the right dose at the right time’’ [5]. The U.S. Genomics and Personalized Medicine Act 2006 explains the term more broadly, namely, as “an application of genomic and molecular data to better target the delivery of healthcare, facilitate the discovery and clinical testing of new products, and help determine patient’s predisposition to a particular disease or condition’’ [6].

In international comparison, the development and market introduction of Personalized Medicine products and services took place in the U.S. particularly fast. In this country, the offer of personalized drugs, treatments and diagnostics has increased from 13 in 2006 to 113 in 2014 [7].

In 2015, the President of the United States, Barack Obama, announced the “Precision Medicine Initiative” (PMI). The objective of this initiative is to gain momentum of Precision Medicine which is seen as a synonym of Personalized Medicine. Its budget for 2016 is $215 million. Of those, $130 million is allocated to NIH and $70 million to the National Cancer Institute [8].

Not only in the U.S. but also in Germany, Personalized Medicine (in this country better known as Individualized Medicine (IM)) has gained more attention. By the end of 2010 the German Federal Ministry of Education and Research (BMBF) named Personalized Medicine to be one of the six priorities and introduced the Action Plan called “Individualized Medicine: a New Way in Research and Healthcare” [9]. Since then the German BMBF supported the validation of biomarkers and implementation of personalized therapies into the clinical trials with over €40 million [10]. Together with 27 partners from 14 countries, the German BMBF also participates in the European PM project called “Personalized Medicine 2020 and Beyond” [11]. The largest PM project in Germany—the German National Cohort (GNC)—was launched in 2013. This project is a nationwide, long-term study with an overall duration of 25–30 years and €210 million budget for the first 10 years [12]. Though, despite these projects, the Personalized Medicine offer in Germany is still significantly smaller than in the U.S., as, until 2016, only 47 Personalized Medicine relevant active substances [13], 30 personalized drugs, [14] have been approved.

As the largest country-specific PM projects in Europe outside Germany, the projects in the UK, France and Norway can be named. In the UK, in 2011, the Stratified Medicine Initiative was launched, with a £60 million budget. It focuses on patient cohorts and biomarker, genotypic and phenotypic analyses [15]. In 2012 Genomics England launched the so-called 100,000 Genomes Project. The goal is to collect genome sequencing of 100,000 NHS patients by 2017 [16]. In France there are the Integrated Cancer Research Centers projects (SIRIC) and the French National Alliance for Life Sciences and Health projects (Aviesan). The SIRIC projects were designated to find new opportunities for cancer treatment [17] and Aviesan—to bring together all players relevant for biomarkers and companion diagnostics [18]. In Norway, the large, long-term HUNT study combines genetic data with clinical records, cancer, stroke and death registries [19].

Among the most significant Europe-wide PM projects is the Innovative Medicine Initiative (IMI-2) supported by the European Medicines Agency (EMA). (This initiative started in 2014 and is planned to run 10 years [20]). The other study, the European Prospective Investigation into Cancer and Nutrition (EPIC), is a large cohort study with 521,000 participants from 10 European countries. (It incorporates genetic and biomarker investigations and is planned to run 15 years [21]). Furthermore, there is the ERA-Net on rare diseases project (E-Rare-3), including 25 institutions from 17 European, Associated and non-European countries. (The project focuses on gene-, cell- and pharmaceutical therapy for rare diseases and is planned for five years (2015–2019) [22].

In general, as for now, despite high expectation in the U.S., Germany and other countries, Personalized Medicine has not become a medical standard for many conditions [23,24] and there is an ongoing discussion as to whether Personalized Medicine is a hope or hype [25,26,27,28]. In fact, Personalized Medicine is now widely used only in oncology [2,29,30], particularly for treatment of melanoma, metastatic lung, breast, or brain cancer and leukemia. The concept has also improved the drug dosing in the field of psychiatry, coronary and peripheral artery diseases, as well as inflammatory bowel diseases [2]. The further areas where many patients could benefit from personalized drugs are pneumology, endocrinology and rheumatology [31].

From the medical side, there have been already a number of studies which have evaluated the advantages of biomarkers, personalized drugs and therapies [32]. As just a few studies show the public and physician attitude to Personalized Medicine aspects (see examples below), and there is no international comparison of those, we decided to perform our own study and provide more evidence in this field. The main aim of our research was to find out whether in the U.S., as the first country which introduced Personalized Medicine [1], and in Germany, there are significant differences regarding the public and physician awareness, acceptance and use of Personalized Medicine, as well as attitude to PM reimbursement, genetic privacy and legal protection. The choice of these aspects was based on the opinion of the U.S. Personalized Medicine Coalition (one of the major PM driving forces in the U.S.), seeing them as the first preconditions for the PM wide implementation into the medical standard [31].

Some of the Personalized Medicine aspects which are a part of our research have been also the focus of several separate surveys, conducted mainly in the U.S. Among physician surveys was Medco National Pharmacogenomics Physician Survey 2008, which discovered physician use of genetic information in daily practice [33]. The U.S. Nationwide Survey for Adoption of Pharmacogenomic Testing by U.S. Physicians 2012 provided the data: how many physicians were informed about pharmacogenomic tests, how many ordered them and what the early adoption of pharmacogenomic tests depended on [34].

Among the public surveys in Germany there was the Forsa study in 2012 on behalf of the BMBF, called “Individualized Medicine – Public Thoughts, Hopes and Fears”. This study described the percentage of the public who have heard about the concept, with the split on gender, age and education level [35]. “The PACE Cancer Perceptions Index: Public Opinion Survey of Cancer Knowledge and Attitudes” was conducted in six countries (France, Germany, Italy, Japan, the U.S. and the UK) in 2012. The survey focused on Personalized Medicine awareness, public perceived cost-saving from PM and the responsibility of covering the PM costs [36]. The study “European Patients’ View on the Responsiveness of Health Systems and Healthcare Providers” was performed in eight European countries (Germany, Italy, Poland, Slovenia, Spain, Sweden, Switzerland and the UK) in 2005. Among other topics, the study aimed to learn about expectations on patient involvement in the treatment decisions [37]. The U.S. Consumers’ Views of Pharmacogenetics 2009 [38], Cogent Genomics, Attitudes & Trends study (CGAT™) 2010 [39], and Formative Research on Perceptions of Biobanking in 2012 [40], described public concerns about genetic data storage, privacy and access. One of the newest studies, the U.S. Public Opinion about Personalized Medicine conducted by the Personalized Medicine Coalition in 2014, explored opinions on reimbursement of personalized tests and treatments [41].

The named PM studies provided important insights, but had some limitations, as they were mainly focused on one particular PM aspect, took the perspective of one healthcare stakeholder and were limited to the situation in one particular country, in most cases the U.S. We believe that our study, based on the surveys in Pennsylvania (U.S.) and Bavaria (Germany), is unique to the field, as the collected data provide comparable physician and public opinions on many PM aspects in two countries.

## 2. Methodology

Our surveys among physicians and the public in Pennsylvania (U.S.) and Bavaria (Germany) were conducted in the time period between May, 2011 and November, 2013. In order to ensure responders’ honest and better comparable answers both of our surveys were anonymous and questionnaire-based.

Searching for Pennsylvanian physicians who could participate in the survey, we used the free accessible physician registries (www.pennmedicine.org, www.yellowbook.com, www.mercyhealth.org, www.templehealth.org). In Germany, we used the database of the Bavarian State Medical Association (www.arzt-bayern.de) and MedKolleg (www.med-kolleg.de/arzt/search.html). For the selection, we used a random principle, favoring those physicians with detailed physician profiles and contact addresses. Physicians who were chosen and later asked to participate in the survey specialized in the medical fields where we expected Personalized Medicine in the future to be a better alternative in comparison to the standard medicine, namely in oncology, psychiatry, internal medicine, diabetes, endocrinology, allergy-immunology, general medicine and pediatrics. The chosen physicians were working in the inpatient and/or outpatient sector, namely in hospitals, clinics, medical centers and medical practices.

Because of the high workload in the hospitals and other medical facilities, and not having the ability to get a physician appointment for filling in a questionnaire, physicians were contacted via letter. In each letter there was a questionnaire with a standard list of terms as well as a stamped envelope with a return address. In total, 350 invitation letters were sent out in Pennsylvania and later the same amount in Bavaria. Of those, in Pennsylvania 57 letters with filled-in questionnaires were returned, and in Bavaria, 90. This shows the physician response rate of 16% in Pennsylvania and 26% in Bavaria. The possible explanation for the relatively low response rate could be physician lack of time, lack of PM knowledge, or a negative attitude to the surveys in general.

The public survey participants were contacted in person. They were over 20 years old and had a certain level of education. In the survey, all social groups participated. Among them were students, university lecturers, employees, non-employees and retirees. Students and employees made up the majority. In Pennsylvania students were contacted on the campus of six universities: Philadelphia University, Temple University, Thomas Jefferson University, Villanova University, College of Medicine in Drexel University and West Chester University. In Bavaria, students were contacted on the university campus in nine cities (e.g., Erlangen, Bayreuth, Munich, *etc*.). Participating employees were mainly passersby in the university campus or clinics, employees of the universities, representatives of the healthcare branch, as well as a few pharmacists.

Survey participation refusals among the public in the U.S. and Germany were under 10%, mainly taking place within the introduction phase. Those refusals were mostly explained by the lack of time or not having the ability to give a comment on Personalized Medicine. The total high response rate among the public can be explained by the high interest in the PM topic as well as there being a favorable place and point of time for filling in the questionnaire.

For the surveys, we used two completely self-designed questionnaires, one for physicians and another for the public (see Supplementary Materials). Physician questionnaires in Pennsylvania and Bavaria were identical. The same is true for the public questionnaires. Questionnaires in Pennsylvania were available in English, and in Bavaria in German. During the translation there were just a few text adjustments, e.g., replacing “Medicare/Medicaid” with “governmental funds” for the German questionnaire. The questionnaires were developed based on the previous literature review and available in paper and electronic form.

While designing our questionnaires we pre-defined some hypotheses which we wanted to prove. We assumed that Personalized Medicine is enhanced by the problematic of adverse drug side effects. We predicted that healthcare participants in the U.S. wish more patients’ involvement in the decisions about their medical treatment, as is the case in Germany. As Personalized Medicine comes from the U.S., we believed that the concept as a whole, as well as personalized drugs and pharmacogenetic tests in particular, are better known, accepted and used among the public and physicians in the U.S. than in Germany. Additionally, we assumed that the public PM acceptance depends on age, gender and health insurance availability and its coverage. We expected physicians working at the hospitals to be better informed about Personalized Medicine. It was suggested that less than 10% of physicians have sufficient experience in analyzing the results of genetic and pharmacogenetic tests today, and that physicians’ willingness to get trained on PM depends on their age. Physicians having Electronic Health Records and family medical histories of their patients were seen to be more likely to accept Personalized Medicine.

We predicted that among the public and physicians there is a strong concern about genetic data use. In this context we expected that healthcare participants, particularly in the U.S., aim to standardize Personalized Medicine regulations for test ordering and involvement of the physician in the test validation. We needed to find out whether the public and physicians wish genetic data to be managed by one governmental, or several private databases. With respect to the growing healthcare online market we expected the U.S. public to like buying individualized drugs and tests online and want this offer to increase in the future. For PM financial aspects we wanted to know whether physicians and the public expect Personalized Medicine to be more effective and money-saving than a standard medicine.

In the questionnaire, within a short introduction on the first page, the survey participants were informed that our survey takes on average 10–15 min, but has no strict time limitations. Both physicians and the public were also informed that before filling in the questionnaire there is a need to read the list of PM terms, including personalized medicine, genetic test, personalized drug, personalized therapy, pharmacogenetic test, protein test and direct-to-consumer test (see Explanation of Terms at the end of this article). While filling in the questionnaire, the participants could always take a look at the explanation of these terms. Additional questions were not answered in order not to influence the survey results.

At the beginning of the questionnaire, we asked physicians and the public whether they have heard about personalized drug, therapy, and personalized medicine, as well as genetic, pharmacogenetic, and protein tests. These questions were very general, mainly referring to the term itself. Providing the answers, survey participants could choose between “yes” and “no”.

For the most of the questions, a Likert five-point rating scale (strongly disagree (1), disagree (2), neither agree nor disagree (3), agree (4), strongly agree (5)) was used. For better comparability of physician and the public answers, most of the questions were identical and grouped into three blocks: (1) general questions, (2) questions about data security, (3) financial questions. The difference was only in additional questions for physicians regarding their prescription of personalized drugs, therapies and genetic, pharmacogenetic and protein tests in general, as well as their experience in analyzing these tests. Asking physicians about their PM experience, we have not predefined some particular fields of experience. Physicians had an opportunity to give their statements according to their individual understanding of PM experience. The further physician-specific questions were related to physician availability of the patients’ Electronic Health Records (EHR), as well as intention to take Personalized Medicine training and readiness to pay for it. The public-specific question was regarding readiness to buy personalized drugs and tests on the internet. These additional questions for physicians and the public were also to be answered on the 5-point scale.

Personalized Medicine acceptance among the public and physicians was measured based on their answers whether “with Personalized Medicine approach it will be possible to deliver better medical care”, and whether “Personalized Medicine is a medicine of the future”. As the Spearman correlation between these two variables was low, for further analysis we used only perceived PM potential to deliver better medical care as a sign for the public and physician PM acceptance.

All survey participants were asked to provide an acceptable price difference between a daily dose of a standard and personalized drug as well as acceptable price difference between a treatment with a standard and personalized therapy per day. Answers could be given in percentage or as an absolute amount. The question was referring to the total general price differences for the whole society, disregarding which drug or therapy and who could pay for it. It was our intention to keep these questions very general and in this way to find out whether the survey participants tend to accept rather high or very low PM prices. The answers for these questions were not predefined and afterwards results were put in the groups like “$1–50”, “$51–100”, “$101–200” and “$201–500”. For the answers in percentage, the groups were created in 10% steps up to 50% (e.g., “1%–10%”, “41%–50%”). The last group was “51%–100%”. Both physicians and the public were additionally asked to estimate an acceptable price for genetic, pharmacogenetic and protein tests. Only physicians were asked to give their assumptions about the possible place where pharmacogenetic, genetic and protein test data are stored now. These free text answers were afterwards grouped in the categories “patient record”, “patient electronic record”, “laboratory”, and “no idea”.

At the end of the public and physician questionnaires there were fields for the general socio-demographic information (e.g., age and gender). Only in the public questionnaire there were additional fields for the survey participant status (e.g., student, university lecturer, employee, *etc.*), diseases (cancer, diabetes, *etc.*), responsibility of covering the healthcare costs and maximal expenses coverage provided by the health insurances. Only physicians were asked about their specialty and place of work. Multiple answers were possible.

Not legible answers, answers like “do not know” and “no idea” were seen as missing values. In total, in the questionnaire standard blocks with general, financial and data security questions on the 5-point-scale, there were only a few missing values. Of those, most of the missing values were in the German public survey for the question regarding governmental support of Personalized Medicine.

Within the open questions, the public participants very often experienced difficulties in providing PM associations, or estimation for an acceptable price difference between standard and personalized drugs, standard and personalized therapies, as well as an acceptable price for PM tests. The general reason for inability to provide price estimations could be a lack of survey participants’ own use of Personalized Medicine or inability to compare standard medicine with Personalized Medicine.

For the socio-demographics of survey participants, there were just a few missing values for gender and age. The possible reason for the missing values is the survey participant forgetting or not willing to provide the information.

The survey results were analyzed by means of descriptive and non-parametric statistic methods using IBM SPSS Statistics software. Within descriptive methods, we often used percentage values, summarizing “agree” and “strongly agree” responses to calculate the share of positive statements. Correlation coefficients (r) were calculated using the Spearman correlation model. The correlation level was considered significant when *p* < 0.05. The use of ANOVA analysis was not possible as Likert-based variables are ordinal scaled. As the data were not normally distributed, the most commonly used were non-parametric statistic methods, such as the *U*-test of Mann and Whitney, the *H*-test of Kruskal and Wallis, with provision of mean rank (mr), grouped median (m) and error probability (*p*). The analyzed effect was considered significant when *p* < 0.05.

## 3. Results

### 3.1. Description of Sample

In Pennsylvania, 155 public representatives (47% male, 53% female) and 57 physicians (61% male, 39% female) participated in the survey. Employees (46%) and students (41%) belonged to the best represented public groups. Their most common age group was 20–30 (Table 1). There were only a small number of the public participants who stated to be under the treatment of serious illnesses relevant for Personalized Medicine—like cancer, diabetes, asthma, depression, *etc*. In the physician survey, physicians in the age between 31 and 40 were the best represented group, which made up about 32% of all participants (see Table 2). In terms of specialization, the majority of physicians were practicing in general medicine, internal medicine and oncology (see Table 3). Some physicians worked at several medical facilities. Of 57 responding physicians, in total 35% of physicians named hospital to be among their places of work, 7% named clinic, 7% medical center, 68% medical practice and 3.5% other medical facility.

In Bavaria, 300 public representatives (48% male, 52% female) and 90 physicians (55% male, 45% female) participated. In the public sample, students made 60%, while employees 33%. The most common age was 20–30. Among the public participants, there were only a few who stated to be under the treatment. In the physician survey, the best represented physician group (44%) was in the age between 41 and 50 years. The majority of physicians worked as internists, general practitioners, oncologists, or pathologists (see Table 3). Almost all physicians worked for only one medical facility. Of all responding physicians, 41% worked at hospital, 5.6% in a medical center, 53% medical practice and 3.5% in other medical facility.

### 3.2. Personalized Medicine Awareness, Acceptance and Use

In Pennsylvania, almost all participants (94% public and 95% physicians) had concerns about adverse drug side effects. In Bavaria only a half (namely, 51% public and 65% physicians) shared these concerns.

In comparison to Pennsylvania, where 86% of the public representatives and 71% of physicians wished to increase patients’ involvement in the decisions about their medical treatment, in Bavaria these were 65% of the public representatives and 32% of physicians.

**Figure 1:**
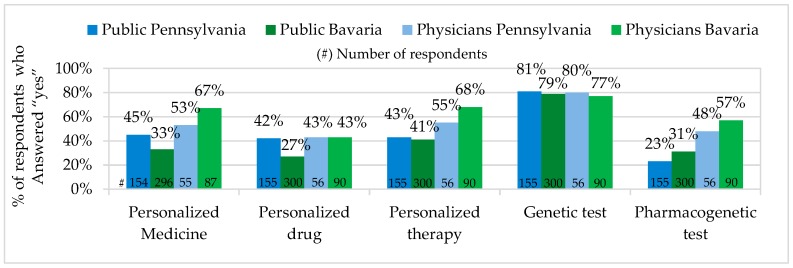
Personalized Medicine Awareness.

In Pennsylvania, the percentage of the public representatives who have heard about Personalized Medicine was higher, while the percentage of physicians was lower, than in Bavaria. In Pennsylvania more public representatives have heard the term personalized drugs. The percentage of physicians who have heard of personalized drugs in Pennsylvania and Bavaria seemed to be on the same level. The large majority of all survey participants stated to have heard about genetic testing, while pharmacogenetic testing seemed to be better known among physicians and the public in Bavaria (see Figure 1).

According to the *U*-test of Mann and Whitney, in our survey, physicians working at the hospitals of Pennsylvania and Bavaria were slightly less informed about Personalized Medicine than physicians in other facilities. This effect was in both states not significant (Pennsylvania *p* = 0.127/ Bavaria *p* = 0.149) and cannot be taken as describing physician populations in Pennsylvania and Bavaria.

**Figure 2:**
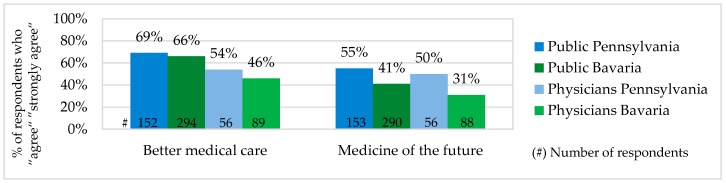
Personalized Medicine Acceptance.

The public and physician acceptance of Personalized Medicine was measured based on their evaluation of PM potential to deliver better medical care and to become a medicine of the future. According to the both evaluations, PM acceptance among physicians and the public in Pennsylvania was slightly higher than in Bavaria. Comparing answers of the public and physicians, it is to mention that in both states PM acceptance of the public was slightly higher (see Figure 2). The public and physicians assumed that PM potential to deliver better medical care and to become a medicine of the future were not related to each other. There were a number of survey participants who have seen PM as a better medical option but did not expect it to become a standard in the future.

As shown in Table 4, according to the *U*-Test of Mann and Whitney as well as the *H*-Test of Kruskal and Wallis, in Pennsylvania and Bavaria, age and gender had no significant influence on Personalized Medicine acceptance among physicians. Age, gender, health insurance availability and its coverage had no influence on PM acceptance by the public (*p* > 0.05). On the table, mean rank (mr) and grouped median (m) show which group of responders had the highest (respectively the lowest) PM acceptance. For example, male physicians in Bavaria (mr = 43; m = 3.3) had slightly lower PM acceptance than female (mr = 46.4; m = 3.5), but in Pennsylvania this effect was *vice versa*. Physicians with the highest PM acceptance in Pennsylvania belonged to the age group 51–60 (m = 3.9), in Bavaria 61–70 (m = 3.8). Among the public in Pennsylvania, the highest PM acceptance had responders in the age group 61–70 (m = 4.0) and in Bavaria >70 (m = 4.3).

In both states (PA: based on 102 responses out of 143 / BY: 154 responses out of 272), the majority of the public representatives stated to have insurance coverage of 80%. PM acceptance of these groups was on a similar high level (PA: m = 3.9 / BY: m = 3.8). PM acceptance among the public with private insurance in Pennsylvania (m = 3.8) and Bavaria (m = 3.7) seemed also to be on a very similar level.

In our survey in Pennsylvania, the stronger a physician agreed to have Electronic Health Record (EHR) or family medical history of his patients, the lower was his PM acceptance. The PM acceptance grouped median of those physicians who strongly agreed to have EHR or family medical history was 3.4, of those who disagreed 3.9. In Bavaria, the trend was the same but without constant dependency. Those physicians who strongly agreed to have EHR showed the lowest PM acceptance (m = 2.8), and those who strongly disagreed—the highest PM acceptance (m = 3.5). Though, according to the *H*-test of Kruskal and Wallis, the influence of EHR availability on PM acceptance in both cases was not statistically significant. So, in the population, EHR has no influence on physician PM acceptance.

Physicians in Pennsylvania and Bavaria stated to have a very similar experience in analyzing the results of genetic and pharmacogenetic tests. Genetic tests could be analyzed by 35% of Pennsylvanian and 29% of Bavarian physicians (PA: 57 / BY: 82 answers), while pharmacogenetic tests in both states could be analyzed by 19% of physicians (PA: 57 / BY: 86 answers).

**Figure 3:**
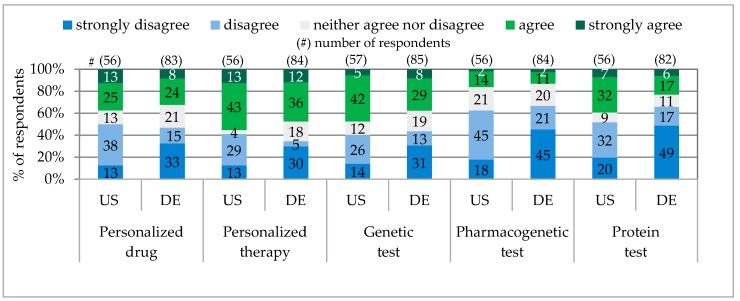
Physicians Prescribing and Advising Personalized Medicine.

According to their own statements, a very similar percentage of physicians in Pennsylvania and Bavaria advised and prescribed personalized drugs, e.g., PA 38% / BY 32%. (A similar receiving of personalized drug prescription and advice in both states was also communicated by the public, e.g., PA 7% / BY 4%). In comparison, in Pennsylvania 10% more physicians advised and prescribed genetic tests, 3% pharmacogenetic tests and 16% protein tests (see Figure 3).

The correlation between physician experience of analyzing the results of pharmacogenetic tests and his prescription and advice of personalized drugs was low (PA: r = 0.468**; *p* = 0.000; N = 56 / BY: r = 0.461**; *p* = 0.000; N = 83).

In Pennsylvania and Bavaria, there was a big difference between the number of physicians planning to get trained on Personalized Medicine. In Pennsylvania they were about 33%, while in Bavaria only 2%. In Pennsylvania there was a middle Spearman correlation between physicians having heard of pharmacogenetic testing and the wish to get trained on PM (r = 0.531**; *p* = 0.000; N = 56), and low correlation between physicians having heard of pharmacogenetic testing and the wish to pay for PM training (r = 0.379**; *p* = 0.004; N = 55). In Germany, these correlations were extremely low and not significant.

In our sample, only by chance physician willingness to get trained on Personalized Medicine was increasing with higher age (e.g., in Pennsylvania age group 31–40 had grouped median 2.7, while group >70 had grouped median 3.5). Based on the Kruskal-Wallis-test in the population, physician willingness to get trained on PM was not dependent on age (Pennsylvania *p* = 0.493 / Bavaria *p* = 0.881). Physician willingness to pay for the training was also not dependent on age. However, there was a very significant dependency between willing to get PM training and readiness to pay for it (PA: r = 0.648**; *p* = 0.000; N = 56 / BY: r = 0.667**; *p* = 0.000; N = 85).

### 3.3. Genetic Privacy and Legal Protection

In Bavaria, the wish to standardize Personalized Medicine regulations was much stronger than in Pennsylvania. Approximately 84% of the Bavarian public aimed to standardize regulations for test ordering and 87% for physician involvement in evaluation of genetic information. Almost all Bavarian physicians supported both of these PM regulation types. In Pennsylvania, 29% fewer public and 21% fewer physicians wanted to standardize test ordering, while 25% fewer public and 11% fewer physicians, to standardize regulations for physician involvement in evaluation of genetic information. In all interviewed groups, there has been a strong correlation between the wish to standardize PM regulations on test ordering and physician involvement in genetic data evaluation (e.g., PA public: r = 0.795**; *p* = 0.000; N = 152; PA physicians: r = 0.726; *p* = 0.000; N = 56).

**Figure 4:**
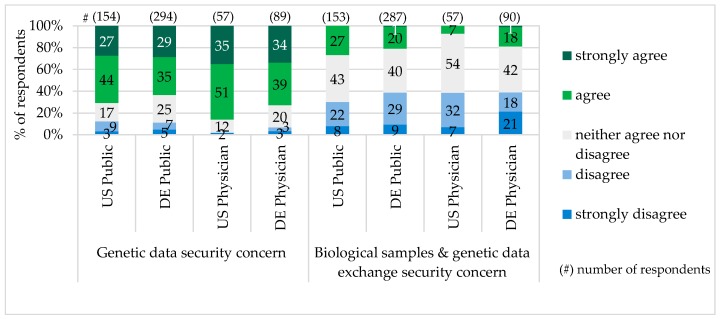
Concern about Genetic Data Security.

The majority of survey participants characterized exchange of biological samples and genetic data in their country as not secure and worried about the genetic data misuse. Particularly in Pennsylvania, more public representatives and physicians had concerns about the genetic data security (see Figure 4).

**Figure 5:**
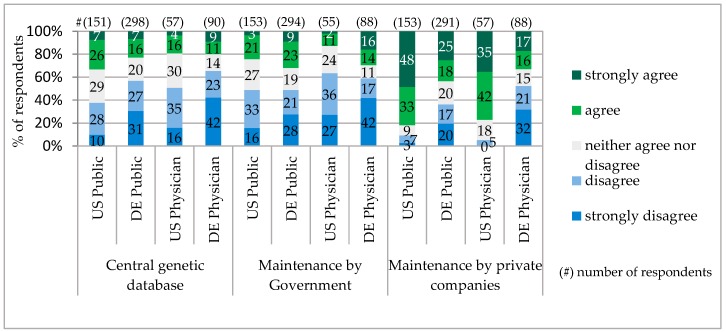
Genetic Database Models.

For the storage of genetic information, the majority of respondents in Bavaria as well as in Pennsylvania did not accept one central database. Among the public, the supporters of a central database were only 33% in Pennsylvania and in Bavaria, 23%. Very similar low acceptance among the public and physicians gained an option of genetic data maintenance by the Government. The majority of the public and physicians in Pennsylvania preferred genetic data to be managed by the private companies. The Bavarian public also shared this opinion, though with significantly fewer supporters. In contrast, the majority of Bavarian physicians were against giving private companies the right to manage the genetic data (Figure 5).

The access to the genetic data was seen as a critical topic. Almost all public and physician survey participants in both countries were against giving employers an opportunity to get the genetic data of their employees. Health insurers were also not accepted to get the access. In Pennsylvania only 18% of the public and 9% of physicians, while in Bavaria 8% of the public and 4% of physicians, would grant health insurances access to the genetic database. Of all possible users of genetic data, the patient was seen as the most acceptable. About 95% of the public in Pennsylvania and 81% in Bavaria were interested in getting access to their genetic information. In Pennsylvania, 90%, while in Bavaria, 68%, of interviewed physicians were willing to grant this access.

There were different opinions regarding increasing or prohibiting Personalized Medicine offers on the internet. In Pennsylvania only 13% of the public liked to buy personalized drugs and tests on the internet, about 47% supported increase of this offer online, while only 15% would prohibit it. In Bavaria, almost all public representatives would not like to buy personalized drugs and tests on the internet. About 14% of the public representatives (three times fewer than in Pennsylvania) would increase the offer online and 26% prohibit it. Half of the physicians in Pennsylvania and Bavaria would completely prohibit the PM offer online.

There were very few physician answers regarding the storage place of the data from pharmacogenetic, genetic, and protein tests today. In Bavaria, of 38 provided answers, “patient record” was the most common answer. It was provided by 11 physicians (29%). Six physicians (16%) named “patient electronic record” and 5 physicians (13%) “laboratory”. Seven responders (18%) provided the answer “no idea”. Among further estimations, were local practice server, internet, patient, *etc*. In Pennsylvania, for this question there were 23 missing values and 14 answers “no idea”. Of 20 available assumptions, 12 were for “patient record” in general, another 8 for “patient electronic record”.

### 3.4. Savings and Reimbursement

In both states, Pennsylvania and Bavaria, approximately the same public majority (60%) shared the opinion that personalized drugs are more effective than standard drugs. Physician expectations differed. In Pennsylvania the majority of physicians (43%) have seen personalized drugs to be more effective, while most of Bavarian physicians (24%) did not share this point of view.

The conviction that personalized drugs cause fewer adverse side effects shared 38% of the public in Pennsylvania and 50% in Bavaria. The majority of physicians (32%) in Pennsylvania expected adverse side effects of personalized drugs to be fewer, while in Bavaria the majority (30%) did not evaluate this expectation as realistic.

A very similar percentage of the public representatives in Pennsylvania (49%) and Bavaria (44%) as well as the majority of physicians in Pennsylvania (28%) hoped that Personalized Medicine can reduce hospitalization days. The majority of physicians in Bavaria (47%) were skeptical about it.

**Figure 6:**
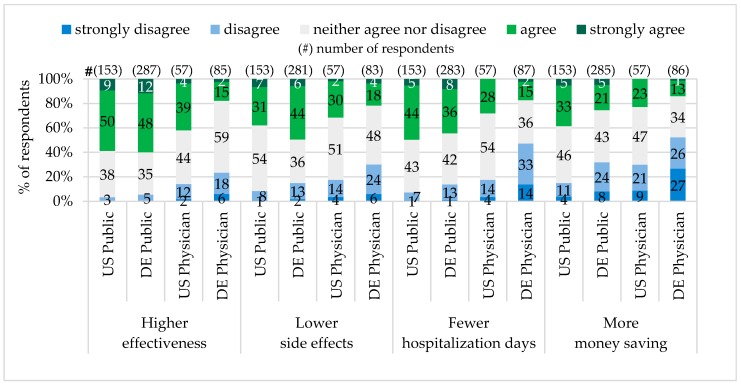
Perceived Personalized Drug Advantages.

The majority of the public in Pennsylvania (38%) have seen Personalized Medicine as a money saving concept for the society in total. In contrast, the majority of Pennsylvanian physicians (30%), the Bavarian public (32%) and physicians (53%) did not believe that Personalized Medicine can be money saving (see Figure 6).

**Figure 7:**
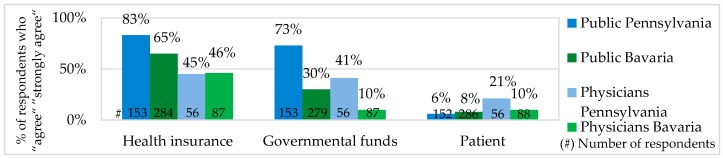
Healthcare Stakeholder Responsible to Cover Personalized Medicine Costs.

Most of the public and physicians in Bavaria and Pennsylvania agreed that the costs for Personalized Medicine should not be covered by patients themselves. In Bavaria, 71% of the public was against paying for Personalized Medicine out-of-pocket, about 65% considered health insurances and 30% German governmental funds to be responsible for the PM costs. In Pennsylvania, 63% of the public were against out-of-pocket payments, 83% expected health insurances and 73% Medicare and Medicaid (U.S. governmental healthcare funds) to cover the costs (see Figure 7).

According to the high Spearman correlation values, Pennsylvanian physicians and the public broadly shared the opinion that if Personalized Medicine would be covered by Medicare and Medicaid it should be also covered by health insurances (PA physicians: r = 0.940**; *p* = 0.000; N = 56 / PA public r = 0.745**; *p* = 0.000; N = 153).

The public and physician acceptable price difference between a daily dose of a standard and a personalized drug as well as a standard and personalized therapy were as following:

Drug: In Pennsylvania 68 public representatives provided data for the drug price difference as an absolute amount. Of those, 15% did not accept any price difference, only 12% would accept $100 and more. The other 73% accepted the drug price difference between $1–50. In Bavaria, 215 public participants provided their price estimation for personalized drug in percentage. Four people did not accept any price difference. Of those who gave an answer in percentage, 27% agreed with the price difference of 11%–20%. Almost the same number of survey participants (22%) accepted a price difference of 1%–10% and 21%–30%. About 9% supported a price difference between 51%–100%.

Therapy: The Pennsylvanian public opinion about the therapy price difference was represented by 63 participants who gave their estimation in absolute numbers. Of those, 21% did not accept any price difference. About 25% have seen price difference $1–10 (another 25% the price difference $11–50) as the most acceptable. One hundred dollars and more would be accepted by 29% of the public. In Bavaria, 205 public representatives answered in percentage. Approximately 24% of them considered a therapy price difference between 21%–30% as the most acceptable. Almost the same number of survey participants (~23%) named the difference of 1%–10% and 11%–20%. The price difference of 31%–40% was accepted by 4% of respondents. About 17% accepted the range of 41%–50%. Only 10% of the public representatives would tolerate a price difference of 51%–100%.

For pharmacogenetic tests, the majority of the Pennsylvanian (40%) and Bavarian (49%) public as well as Bavarian physicians (46%) have seen the price $/€1–50 as the most acceptable. The price named by the majority of Pennsylvanian physicians (36%) was much higher, namely $201–500.

In general there was a very high correlation between acceptable price for genetic and pharmacogenetic tests (e.g., PA phys: r = 0.931**; *p* = 0.000; N = 14 / BY phys: r = 0.736**; *p* = 0.000; N = 28).

## 4. Discussion

According to our assessment, the concerns about adverse drug side effects and the wish to increase patient involvement in medical decisions have a potential to speed up the wide spread of Personalized Medicine in the U.S. and Germany. In our survey, almost all interviewed physicians and the public representatives in Pennsylvanian and the majority in Bavaria, had concerns about adverse drug side effects. The wish to increase patient involvement in medical decisions was much stronger among the public and physicians in Pennsylvania. In Bavaria, the majority of the public representatives also supported the idea but the number of physicians supporting increase of patient involvement and those being against it was almost the same. Comparing our figure on desired patient involvement in Bavaria (65%) with the figure of “European Patients’ View on the Responsiveness of Health Systems and Healthcare Providers” study 2005 (where 75% of European patients, particularly of the younger age, wished to play a more active role in a treatment decision-making) [37], it is worth mentioning that our Bavarian figure was under the European average.

Our survey results have shown that the majority of the public in Pennsylvania and Bavaria have heard of personalized therapy and genetic tests. Personalized Medicine, pharmacogenetic tests and personalized drugs were not widely known among the public. The majority of physicians in both states have heard of Personalized Medicine, personalized therapy, genetic tests and pharmacogenetic tests. Physicians who have heard of personalized drugs did not make up the majority.

Personalized Medicine: Unexpectedly, the percentage of physicians who have heard about Personalized Medicine was a little lower (PA 53% / BY 67%) in Pennsylvania, but, as predicted, the corresponding percentage of the public was higher in Pennsylvania than in Bavaria (PA 45%/BY 33%). In comparison, our figure for the Pennsylvanian public was very similar to the one of the PACE study, 2012 (48%) [36], and little higher than the one from the U.S. Public Opinion Study (PMC), 2014 (38%) [41]. The explanation for the difference between the figures in the PMC study, 2014, and our study could be the fact that in our survey we from the very beginning provided the definition of the term, Personalized Medicine. In the PMC study, the survey participants received the explanation of the term only after answering the question whether they have heard of the concept. In Bavaria, our results in the public survey corresponded precisely to the results of the joint study of the Forsa and the German BMBF called “Individualized Medicine—Public Thoughts, Hopes and Fears”, which showed that in Germany in 2012, out of 1000 public survey participants, about 65% have never heard the term “Individualized Medicine” [35]. This evaluation was also confirmed by the German BMBF in 2013 by saying that in Germany there is a big interest in Individualized Medicine, but the knowledge about the concept is relatively limited [9]. In comparison to the PACE study, 2012, the PM awareness among the public in Bavaria (33%) was very similar to the combined average value (34%) of France, Germany, and Italy, and higher than the one in the UK (29%) [36].

According to our results, the majority of the public and physicians showed relatively high Personalized Medicine acceptance, as they believed that Personalized Medicine has a potential to improve the quality of care and become a medicine of the future. However, these two beliefs were not related to each other. There were a number of people who have seen PM as a better medical option but did not believe that it will be a standard in the future. Of all survey groups, physicians in Bavaria had the lowest PM acceptance and the public in Pennsylvania the highest.

In contrast to our hypothesis, the public PM acceptance was not dependent on age, gender, or health insurance availability, or its coverage. Physician PM acceptance was not influenced by age, gender, or Electronic Health Record availability. Physicians working at the hospitals were not better informed about Personalized Medicine. Similar results were received on some of these aspects in other studies. The Forsa study “Individualized Medicine – Public Thoughts, Hopes and Fears” in 2012 provided evidence that there was no correlation between age (or education level) and hearing the term Individualized Medicine [35]. (The U.S. Nationwide Survey for Adoption of Pharmacogenomic Testing 2012 showed that physician age and gender were not significant predictors of early adoption of pharmacogenomic testing [34]).

Pharmacogenetic and Genetic Tests: in Pennsylvania and Bavaria there was no big difference between the percentage of the public and physicians who have heard of pharmacogenetic tests. In comparison to Pennsylvania (public 23%/physician 48%), in Bavaria, 8% more public representatives and 9% more physicians stated to have heard of pharmacogenetic tests. Pennsylvanian and Bavarian physicians also showed no significant difference for the pharmacogenetic test prescription and advice. According to physicians’ own statements, only 3% more physicians in Pennsylvania prescribed and advised pharmacogenetic tests (PA 16%/BY 13%). We compared our physician data from Pennsylvania with the U.S. National Survey for Adoption of Pharmacogenomic Testing, 2012, where about 10% of physicians stated that they were informed about pharmacogenomic tests and 13% had ordered them in the previous 6 months [34]. In our survey, five times more physicians have heard of pharmacogenetic tests and about the same percentage prescribed them. The difference between only “hearing about the test” and “being informed about it” was probably the main reason for the different results in the studies.

In our study, there was a low correlation between the physician’s experience of analyzing the results of pharmacogenetic tests and his prescription and advice of personalized drugs. It can be explained by the fact that the physician does not have to be able to analyze the results of pharmacogenetic tests. Laboratory staff describe the results of pharmacogenetic tests very well.

The percentage of those physicians who had PM skills was very similar in Pennsylvania and Bavaria and their number was higher than we originally predicted. In both states, about 19% of physicians stated to be able to analyze pharmacogenetic tests. Genetic tests could be analyzed by 35% of physician responders in Pennsylvania and by 29% in Bavaria. In comparison, our values for genetic data use were higher than the ones from the Medco National Pharmacogenomics Physician Survey, saying that in 2008 only 10% of U.S. physicians thought that they would be able to use genetic information in their daily practice [33]. The possible reasons for the result difference can be deviations in the survey sample design for physician specialty and increase of genetic information use in the last years.

Personalized Drugs: among physicians, exactly the same percentage of representatives (PA 43%/BY 43%) stated to have heard of personalized drugs and approximately the same percentage (PA 38%/BY 33%) to prescribe and advise them. Among the public, 15% more representatives in Pennsylvania (42%) stated that they have heard of personalized drugs and a very similar public percentage in both states (PA 7%/BY 4%) confirmed to receive personalized drugs with prescription and advice. In comparison to the U.S. Public Opinion Study (PMC), 2014, where personalized drugs were recommended to 11% of the public [41], our figure for the Pennsylvanian public was very close. The difference of our figures for personalized drugs advice and prescription provided by physicians and the public can be explained by the fact that personalized drugs are currently prescribed to patients with particular diseases and not for a wide range of conditions.

The relatively low willingness of physicians to prescribe pharmacogenetic tests and personalized drugs might be explained by several factors. Firstly, the reimbursement of these tests is not sufficient to recover the costs. Secondly, physicians do not perceive that there is robust evidence of pharmacogenetic tests demonstrating their clinical utility [42]. Thirdly, standard medical education programs do not address Personalized Medicine sufficiently [33,34,43,44].

Remarkable was the fact that in our survey many more Pennsylvanian physicians (PA 33% *vs.* BY 2%) aimed to get PM training in the near future. In contrast to Pennsylvanian, Bavarian physicians who have heard of pharmacogenetic tests were not seeking PM training. As we found significant dependency between willingness to get PM training and willingness to pay for it, it is likely that the majority of Bavarian physicians were not willing to get PM training because of the possible need to pay for it out-of-pocket.

Savings and Reimbursement: Of all factors influencing the wide use of Personalized Medicine, the treatment costs and their reimbursement can be seen as the most important. Tamoxifen and trastuzumab were among the first drugs routinely reimbursed with companion diagnostic tests [45]. Ten years later, nineteen companion diagnostic tests (mainly for cancer treatments) are required by the FDA [46] and are generally reimbursed by most insurance companies. The reimbursement of the tests which are not required by the FDA depends on the health insurer decision [47]. Large private insurance companies, such as Aetna, United Healthcare and Kaiser Permanente have progressive coverage policies that reimburse identification of high risk populations (e.g., BRCA1/2 test for breast cancer), as well as prediction of the benefits from the therapy (Oncotype test for chemotherapy) [31].

In Germany, about 30 personalized drugs (39 active substances) currently require pharmacogenetic pretests [13,14]. The majority of these drugs and corresponding tests are expected to be reimbursed by the statutory health insurances.

There is a broad expectation that the new trends for the reimbursement decisions often come from the U.S., in particular Medicare, the largest U.S. governmental health insurer. If it decides to cover the costs for particular medical services, American private health insurance companies follow [48] very fast. Currently, Medicare reimburses PM services by using Alternative Payment Models (APMs) within the Medicare Shared Savings Program (MSSP) and the Pioneer Accountable Care Organizations (ACOs) [49]. Among the latest reimbursement changes done by U.S. Medicare, were adjustments of coding and pricing for genomic tests in 2012–2014 [45]. In early 2015, supported by the President Obama Administration, a goal was set to tie 30% of fee-for-service Medicare payments to quality or value by means of Alternative Payment Models by the end of 2016, and to tie 50% by the end of 2018 [49].

Though, despite the efforts, in total, insurance companies in the U.S. and Germany are currently reluctant to reimburse biomarker tests and personalized treatments. The main reason for this is a limited number of studies showing healthcare improvement evidence and money savings [50]. Of 59 cost-utility studies (1998–2011) for different PM tests, most of which were performed in the U.S., about one-fifth showed money savings. The other studies provided evidence for better health, though at higher costs [51].

In Germany, according to the German Federal Ministry of Education and Research (BMBF), Personalized Medicine will be money saving only if prevention and illness early detection will be improved, and only the drugs with high probability of a complete cure will be in use [9]. In our study, most of the participants, including the public and physicians in Pennsylvania, as well as the public in Bavaria, expected personalized drugs to be more effective, to have lower adverse side effects, and to reduce hospitalizations days. However, the total healthcare cost reduction was predicted only by the Pennsylvanian public. On the opposite, the majority of Bavarian physicians were very skeptical. In comparison to the PACE study, 2012, where more than 50% of responders in Europe and the U.S. have seen Personalized Medicine as a cost-saving measure, our figures for the Pennsylvanian (38%) and Bavarian (26%) public were significantly lower [36].

According to the opinion of our public survey participants, there should not be a big price difference between Personalized Medicine and standard medicine. The named acceptable overprice for a personalized drug and therapy was about 30%. For the wide use of Personalized Medicine, it is essential not only to decide for which price PM should be offered but also who should be responsible to cover the costs. In several studies it was a widespread opinion that Personalized Medicine should not be financed mainly by patient out-of-pocket payments. In the PACE study, 2012, about 72% of responders in Europe and the U.S. expected health insurances to cover the PM costs [36]. A very similar result (two-thirds of responders) was received in the PMC study, 2014 [41]. In the U.S. National Survey for Adoption of Pharmacogenomic Testing by Physicians, 2012, the figure was significantly higher. About 90.6% of responders believed that pharmacogenomic testing improves drug effectiveness and health insurances should cover it [34]. In our survey, the PM costs were expected to be covered by health insurances and governmental funds. In Pennsylvania, this wish was stronger than in Bavaria. It can be explained by the U.S. health insurance policies having payment caps and more reimbursement exceptions.

Genetic Privacy and Legal Protection: The second challenge for PM implementation is data security. The concerns about genetic information storage and privacy have been already expressed by the public in the study Consumers’ Views of Pharmacogenetics in 2009 [38] and Formative Research on Perceptions of Biobanking in 2012 [40]. In our study, particularly in Pennsylvania (public 71%/physicians 86%), there was also much concern about genetic data misuse. In comparison to the Cogent Genomics, Attitudes & Trends study with 1000 Americans in 2010, which showed concerns about genetic data misuse among 71% of the U.S. public and 79% of physicians [39], our figure for the public was the same, but for physicians a little higher. In Bavaria, our figure for genetic data security concern of the public (64%) was a little lower than the one (70%) for public general concerns regarding private data on the internet in Germany, provided by Forsa study “Individualized Medicine—Public Thoughts, Hopes and Fears” in 2012 [35].

In our study in Pennsylvania and Bavaria, the majority of physicians and the public did not accept one centralized genetic database managed by the Government. The lack of trust was probably one of the reasons why the national U.S. biobank initiated by the Secretary’s Advisory Committee on Genetic, Health, and Society (SACGHS) in 2007 had no efforts underway [52]. Though, beginning in 2016, a new effort for the U.S. national genetic biobank with one million participants starts, called Cohort Program of Precision Medicine Initiative (PMI). The intention is to collect genetic data, biological samples, diet and lifestyle information [53], as well as to reflect social, economic and ethnic diversity of the U.S. population [8]. We believe that in the future the Cohort Program database will be a good information pool to support medical research, as well as Medicare and Medicaid reimbursement decisions. From the infrastructure point of view it probably will not be accepted as a genetic database for the entire U.S. population. According to our survey results in the U.S., the most acceptable PM infrastructure could be created by many decentralized genetic databases, granting access mainly to the physicians and the patients.

Summary: All in all, the public and physician opinions on Personalized Medicine aspects and preconditions for the wide implementation in Pennsylvania and Bavaria in their main tendency were not as different as we originally expected them to be. The majority of respondents had concerns about adverse drug side effects, wished to increase patient involvement and standardize medical regulations. There was a common opinion that genetic data exchange was not secure, that the offer of personalized drugs and tests online should not be increased and there should not be one centralized genetic data base managed by the Government. The majority of respondents did not support the coverage of PM services by the patient out-of-pocket payments and saw health insurances to be responsible to cover most of the costs. Though, it was essential to ensure that health insurances do not get access to the genetic data. The only one aspect where one group of responders had a completely different opinion in comparison to others was the use of the Electronic Health Record (EHR) for genetic data storage. The majority of participants, including the Pennsylvanian and Bavarian public, as well as Pennsylvanian physicians, have seen EHR as an acceptable source for genetic data storage. Physicians in Bavaria did not share this opinion.

The reluctance of Bavarian physicians to accept EHRs can be mainly explained by their strong data security concerns (which is a common phenomenon in Germany), as well as their modest willingness to provide patients access to the genetic data. In contrast, in the U.S., EHR acceptance is higher, so that the data collected within the national Cohort Program of Precision Medicine Initiative (PMI), 2016, will be linked to EHR and become accessible for the cohort participants [53].

The higher acceptance of EHR among Pennsylvanian physicians can be at least partly explained by a strong support by the U.S. Government and its encouragement to switch to EHR by means of incentives and penalties within Medicare and Medicaid reimbursements (e.g., HITECH, ARRA Act, 2009). Favorable was also the fact that, in comparison to Germany, the U.S. has a different decision-making process for the changes in the healthcare system. In the U.S., EHR was a topic on the U.S. Congress level. (In Germany the decision on EHR is made not only on the state and federal level, but also by healthcare councils and committees, e.g., the National Association of Statutory Health Insurance Funds, National Association for Statutory Health Insurance Physicians).

The general explanation of differences between Personalized Medicine data collected in Pennsylvania (U.S.) and Bavaria (Germany) is challenging. The deviations can be explained by the different healthcare systems, culture and history. The differences in the health insurance characteristics may have some influence, e.g., mainly private insurances with therapy cap limits in the U.S. and mainly statutory health insurances without caps in Germany. Further influencing factors could be more intensive communication of the Human Genome Project in the U.S. and a larger amount of approved personalized drugs and therapies on the U.S. market in comparison to the German market.

Our study does not provide a comprehensive benchmark of the U.S. and German physician and public attitude to Personalized Medicine. The findings only partly apply to the total populations in the U.S. and Germany. Physician low response rate, a relatively small physician and public sample size, and geographical coverage, sample structure with some overrepresentation of young people in the public sample and over- or underrepresentation of physicians with a particular medical specialty in the physician sample can be seen as possible limitations. These limitations could induce some biases in the reported overall results.

For further surveys, it would be of advantage to involve more survey participants, to better balance the social groups in the sample and define more pre-requirements for the survey participants. It would be of advantage to reflect stronger on public diversity with respect to social and economic aspects and, particularly in the U.S., to take in consideration public ethnic diversity. The latter will be addressed within PMI Cohort Program in 2016.

## 5. Conclusions

As the Human Genome Project and the majority of Personalized Medicine studies have been performed in the U.S., one would expect Personalized Medicine awareness, acceptance and use in the U.S. to be much higher than in Germany. Though, the results of our research have shown that awareness, acceptance, and use of Personalized Medicine in the U.S. and Germany were not significantly different. One of the main elements of Personalized Medicine—personalized drug—was not known among the majority of physicians and the public in Pennsylvania and Bavaria. Pharmacogenetic testing was known among the majority of physicians, but not the public. Personalized drugs and therapies in both states were advised and prescribed by a very similar percentage of physicians.

The majority of survey participants among physicians and the public shared an opinion that Personalized Medicine has a potential to improve the quality of care and become a medicine of the future, though most likely at higher costs. Of all survey participants, the total healthcare cost reduction from Personalized Medicine was assumed only by the public in Pennsylvania.

Comparing Personalized Medicine in Pennsylvania and Bavaria, we came to the conclusion that in the U.S. there are some more preconditions for Personalized Medicine faster implementation. Our suggestion is based on the fact that in our survey Pennsylvanian physicians and the public had higher concerns regarding adverse drug side effects and a stronger wish to increase patient involvement in medical decisions. In Pennsylvania, more physicians shared the opinion that personalized drugs are more effective, cause less adverse drug side effects and can ensure money savings. Physicians had a stronger wish to get PM training and were ready to pay for it. They had fewer concerns about the use of the Electronic Health Record for genetic data storage. The majority wished to standardize PM regulations, but this wish was not as strong as in Bavaria.

We assume that in the U.S. and Germany there will be differences not only in the speed of Personalized Medicine implementation but also in its way. As in our U.S. survey, three times more public representatives supported an increase of the PM offer online, we predict that this offer in the U.S. will grow much faster, while in Germany it will stay out of trend for a certain time.

Of all preconditions for Personalized Medicine implementation, the most significant are biomarker research, robust evidence of pharmacogenetic tests demonstrating clinical utility, as well as reimbursement, genetic privacy and legal protection. For biomarker research, it is challenging that as for now for a number of diseases it was impossible to find a definitive biomarker. If a large amount of new disease-related biomarkers will not be discovered in the near future this would mean that Personalized Medicine will stay a niche innovation for a long time.

For reimbursement, it is essential that not only personalized drugs but also pharmacogenetic and other related tests would be covered by health insurances and governmental funds. The first steps in this direction have already been taken. Though, the process of inclusion of personalized drugs and tests in the healthcare reimbursement system is very slow and there is a need for further major adjustments. Because of the high complexity within the reimbursement system, the changes in the future will also take time and can be implemented only step by step.

For genetic privacy and legal protection, it is essential to create regulations which from one side protect against genetic data misuse and at the same time enable public voluntary data provision for research purposes. This issue will be addressed by the national PMI Cohort Program supported by the U.S. Government. Within the project preparation phase, a review of the current regulations landscape is prepared to support the data privacy protection of cohort participants. Furthermore, it is intended to work closely with patients, bioethicists and IT specialists, as well as to create jointly the base for the Personalized Medicine regulations. The successful completion of the PMI Cohort Program has a potential to significantly contribute to the wide spread of Personalized Medicine in the near future.

The decisions regarding PM reimbursement and legal concepts in the U.S. and Germany may have their specifics. In the U.S., mainly because of the differences in the 50 U.S. states, and in Germany, as a European country, because of the need to achieve an agreement with other European countries on, at some point, fragmented European regulations and approval authorities.

## Figures and Tables

**Table 1:** Public Age.

(**A**). Pennsylvania (U.S.)							
**Age Bracket**	**20–30**	**31–40**	**41–50**	**51–60**	**61–70**	**>70**	**Missing**
Absolute	76	29	20	21	7	1	1
Relative	49.0%	18.7%	12.9%	13.5%	4.5%	0.7%	0.7%

(**B**). Bavaria (Germany)							
**Age Bracket**	**20–30**	**31–40**	**41–50**	**51–60**	**61–70**	**>70**	**Missing**
Absolute	206	39	22	18	7	3	5
Relative	68.7%	13.0%	7.3%	6.0%	2.3%	1.0%	1.7%

**Table 2:** Physician Age.

(**A**). Pennsylvania (U.S.)							
**Age Bracket**	**20–30**	**31–40**	**41–50**	**51–60**	**61–70**	**>70**	**Missing**
Absolute	0	18	11	15	9	2	2
Relative	0%	31.6%	19.3%	26.3%	15.8%	3.5%	3.5%

(**B**). Bavaria (Germany)							
**Age Bracket**	**20–30**	**31–40**	**41–50**	**51–60**	**61–70**	**>70**	**Missing**
Absolute	0	8	40	26	14	0	2
Relative	0%	8.9%	44.4%	28.9%	15.6%	0%	2.2%

**Table 3:** Physician Medical Specialization.

Pennsylvania	Absolute	Relative	Bavaria	Absolute	Relative
Allergy/Immunology	6	12%	Allergy/Immunology	0	0%
Diabetics	1	2%	Diabetics	2	2%
Endocrinology	4	8%	Endocrinology	1	1%
Gastroenterology	0	0%	Gastroenterology	1	1%
General Medicine	15	30%	General Medicine	25	32%
Internal Medicine	11	22%	Internal Medicine	25	32%
Oncology	7	14%	Oncology	13	17%
Pathology	0	0%	Pathology	9	11%
Pediatrics	1	2%	Pediatrics	0	0%
Psychiatry	3	6%	Psychiatry	2	3%
Surgery	0	0%	Surgery	1	1%
Other	2	4%	Other	0	0%

**Table 4:** Factors to Influence PM Acceptance.

Representative Influence Factor	Public		Physicians	
	Pennsylvania	Bavaria	Pennsylvania	Bavaria
Gender	M: mr = 74.0 (m = 3.8)	M: mr = 149.3 (m = 3.8)	M: mr = 28.4 (m = 3.7)	M: mr = 43.0 (m = 3.3)
	F: mr = 78.8 (m = 3.9)	F: mr = 142.0 (m = 3.7)	F: mr = 24.9 (m = 3.5)	F: mr = 46.4 (m = 3.5)
	*p* = 0.462	*p* = 0.426	*p* = 0.391	*p* = 0.514
Age	20–30: mr = 72.4	20–30: mr = 142.6	20–30: -	20–30: -
	(m = 3.7)	(m = 3.7)		
	31–40: mr = 80.2	31–40: mr = 146.2	31–40: mr = 23.4	31–40: mr = 39.8
	(m = 3.9)	(m = 3.8)	(m = 3.4)	(m = 3.2)
	41–50: mr = 81.1	41–50: mr = 147.4	41–50: mr = 26.6	41–50: mr = 44.4
	(m = 3.9)	(m = 3.8)	(m = 3.6)	(m = 3.4)
	51–60: mr = 81.5	51–60: mr = 164.5	51–60: mr = 33.2	51–60: mr = 40.1
	(m = 3.9)	(m = 4.0)	(m = 3.9)	(m = 3.2)
	61–70: mr = 85	61–70: mr = 130.6	61–70: mr = 29.8	61–70: mr = 52.5
	(m = 4.0)	(m = 3.7)	(m = 3.7)	(m = 3.8)
	>70: mr = 26.5	>70: mr = 195.5	>70: mr = 19.0	>70: -
	(m = 3.0)	(m = 4.3)	(m = 3.0)	
	*p* = 0.616	*p* = 0.727	*p* = 0.338	*p* = 0.443
Health insurance type	State: -	State: mr = 149		
		(m = 3.8)		
	Private: mr = 77.3	Private: mr = 133		
	(m = 3.8)	(m = 3.7)		
	G-fund: mr = 63.4	G-fund: mr = 265		
	(m = 3.6)	(m = 5.0)		
	Myself: mr = 74.8	Myself: mr = 164		
	(m = 3.8)	(m = 4.0)		
	*p* = 0.858	*p* = 0.217		
Health insurance coverage	0%: mr = 71.0	0%: -		
	(m = 3.8)			
	30%: mr = 88.4	30%: mr = 141.1		
	(m = 4.2)	(m = 3.9)		
	50%: mr = 58.6	50%: mr = 128.5		
	(m = 3.5)	(m = 3.7)		
	80%: mr = 73.7	80%: mr = 136.6		
	(m = 3.9)	(m = 3.8)		
	100%: mr = 63.5	100%: mr = 137.6		
	(m = 3.7)	(m = 3.8)		
	*p* = 0.470	*p* = 0.952		

mean rank (mr); grouped median (m); error probability (*p*).

## Supplementary Materials

The survey questionnaires are available online at http://www.mdpi.com/2075-4426/6/2/15/s1.

## Abbreviations

The following abbreviations are used in this manuscript:

ACO: Accountable Care Organizations
APM: Alternative Payment Model
BMBF: German Federal Ministry of Education and Research
CGAT: Cogent Genomics, Attitudes & Trends study
EHR: Electronic Health Record
EMA: European Medicines Agency
EPIC: European Prospective Investigation into Cancer and Nutrition
FDA: U.S. Food and Drug Administration
FFS: Fee-for-Service
HGP: Human Genome Project
IM: Individualized Medicine
IMI: Innovative Medicines Initiative
NHS: National Health Services
PM: Personalized Medicine
PMC: Personalized Medicine Coalition
SACGHS: Secretary’s Advisory Committee on Genetic, Health, and Society
SIRIC: Integrated Cancer Research Centers

## Explanation of Terms

**Direct-to-consumer tests** are directly available to the consumer without the oversight of a clinician, over the counter or from a foreign source via the internet or other means [54].

**Genetic test** is a test to diagnose or determine a person’s genetic risk of developing a condition or disease [55].

**Personalized drug** (genetic drug) is a drug which is chosen and dose-adjusted based on each patient’s molecular diagnosis and genetic makeup [56].

**Personalized Medicine** is the application of genomic and molecular data to better target the delivery of health care, facilitate the discovery and clinical testing of new products and help determine a patient’s predisposition to a particular disease or condition [6].

**Personalized therapy** (genetic therapy) is a therapy arising from genetic research and investigation, including pharmaceuticals, diets, enzyme replacement and gene therapy [57].

**Pharmacogenetic test** is a test intended to identify individual variations in DNA sequence related to drug absorption and disposition or drug action [6].

**Protein test** is a test which measures the amount of proteins, such as albumin, found in a urine sample [58].
