# Supplementary material for: Personalized Medicine in the U.S. and Germany: Awareness, Acceptance, Use and Preconditions for the Wide Implementation into the Medical Standard
Source: J Pers Med. 2016 May 2;6(2):15. doi: 10.3390/jpm6020015 (PMC4932462; doi:10.3390/jpm6020015)
Supplement: Supplementary file 1 [file jpm-06-00015-s001.zip › supplementary/Questionnaires for Physicians and the Public.pdf]

Example: Questionnaire (U.S. Physician)

Have you heard about Personalized Medicine? ☐ Yes ☐ No

What are your associations with Personalized Medicine?

____

____

Have you heard about:
(multiple answers are possible)

Personalized drug ☐ Genetic test ☐ Protein test ☐
Personalized therapy ☐ Pharmacogenetic test ☐

		1<br>Strongly disagree	2<br>Disagree	3<br>Neither agree nor disagree	4<br>Agree	5<br>Strongly agree
	**General Questions**					
1	One-size-fits-all is not the best type of medication					
2	I am concerned about the side effects of drugs					
3	Based on Personalized Medicine approach it will be possible to deliver better medical care					
4	Personalized Medicine will be standard practice in 5 years					
5	Personalized Medicine is the medicine of the future					
6	Socio-political processes in the U.S. encourage the development of Personalized Medicine					
7	Patient involvement in the decisions about their own medical treatment must increase					
8	Amount of direct-to-consumer personalized drugs and genetic tests available online should increase					
9	Direct-to-consumer personalized drugs and genetic tests available online should be prohibited					
10	For genetic tests purchased online, the oversight of a clinician is not necessary					
11	All US States should agree on the regulations regarding ordering of tests					
12	All US States should agree on the regulations regarding the involvement of medical professionals in the evaluation of genetic information					
13	The current U.S. government supports the development of Personalized Medicine					
14	Database(s) holding genetic information should be maintained by government.					
15	Genetic information should be stored in the central database					
16	Database(s) holding genetic information should be private					

		1 Strongly disagree	2 Disagree	3 Neither agree nor disagree	4 Agree	5 Strongly agree
17	All types of laboratory tests should be reviewed by the FDA					
18	EHR (Electronic Health Record) and family medical history are suitable sources for storage of genetic information					
	**Professional Questions**					
19	I prescribe/advice …					
a	Personalized drugs					
b	Personalized therapies					
c	Genetic tests					
d	Pharmacogenetic tests					
e	Protein test					
20	I have experience of analyzing the results of…					
a	Genetic test					
b	Pharmacogenetic test					
c	Protein test					
21	I plan to get some education/training for Personalized Medicine					
22	I am willing to pay for my Personalized Medicine training myself					
23	I have EHR (Electronic Health Records) and family histories of my patients					
	**Financial Questions**					
24	With Personalized Medicine the amount of hospitalization days will be reduced					
25	Personalized drugs are more effective than standard drugs					
26	Side effects of personalized drugs are lower than that of standard drugs					
27	Personalized drugs are less expensive than standard drugs					
28	It is less expensive to develop personalized drugs than standard drugs					
29	Personalized Medicine can help society to save money					
30	Personalized Medicine should be covered by health insurance					
31	Medicare and Medicaid should cover the costs of Personalized Medicine					
32	The patient should pay out-of-pocket to cover the costs of Personalized Medicine					

		1 Strongly disagree	2 Disagree	3 Neither agree nor disagree	4 Agree	5 Strongly agree
	**Questions About Data Security**					
33	Exchange of biological samples and genetic data in the U.S. is secure					
34	Patients should have access to their genetic information					
35	Health insurer for life, disability and long-term care should be allowed to require genetic information					
36	Health insurance companies should have access to genetic information					
37	Employers should have access to genetic information of their employees					
38	I have a concern about the security of genetic data use					
39	I surf the internet every day					

Where is the data about pharmacogenetic, genetic and protein tests stored now?

____

Acceptable price difference between daily dose of standard and personalized drug is: $/%________

Acceptable price difference between treatment with standard and personalized therapy per day is: $/%________

Acceptable price for

Genetic test is: $______________________________

Pharmacogenetic test is: $______________________________

Protein test is: $______________________________

Comment:

____

____

Additional Information:

Gender: Male ☐ Female ☐

Age: 20-30 ☐ 41-50 ☐ 61-70 ☐

31-40 ☐ 51-60 ☐ over 70 ☐

Medical specialization: ______________________________

I am currently working at: hospital ☐ medical centre ☐ other ☐

clinic ☐ medical practice ☐

Example: Questionnaire (U.S. Public)

Have you heard about Personalized Medicine? ☐ Yes ☐ No

What are your associations with Personalized Medicine?

____

____

Have you heard about:
(multiple answers are possible)

Personalized drug ☐
Personalized therapy ☐
Genetic test ☐
Pharmacogenetic test ☐
Protein test ☐

Have you already had:
(multiple answers are possible)

Personalized drug ☐
Personalized therapy ☐
Genetic test ☐
Pharmacogenetic test ☐
Protein test ☐

		1 Strongly disagree	2 Disagree	3 Neither agree nor disagree	4 Agree	5 Strongly agree
	**General Questions**					
1	One-size-fits-all is not the best type of medication					
2	I am concerned about the side effects of drugs					
3	Based on Personalized Medicine approach it will be possible to deliver better medical care					
4	Personalized Medicine will be standard practice in 5 years					
5	Personalized Medicine is the medicine of the future					
6	Socio-political processes in the U.S. encourage the development of Personalized Medicine					
7	Patient involvement in the decisions about their own medical treatment must increase					
8	Amount of direct-to-consumer personalized drugs and genetic tests available online should increase					
9	Direct-to-consumer personalized drugs and genetic tests available online should be prohibited					
10	For genetic tests purchased online, the oversight of a clinician is not necessary					
11	All US States should agree on the regulations regarding ordering of tests					
12	All US States should agree on the regulations regarding the involvement of medical professionals in the evaluation of genetic information					
13	The current U.S. government supports the development of Personalized Medicine					
14	Database(s) holding genetic information should be maintained by government.					

		1<br>Strongly disagree	2<br>Disagree	3<br>Neither agree nor disagree	4<br>Agree	5<br>Strongly agree
15	Genetic information should be stored in the central database					
16	Database(s) holding genetic information should be private					
17	All types of laboratory tests should be reviewed by the FDA					
18	EHR (Electronic Health Record) and family medical history are suitable sources for storage of genetic information					
	**Professional Questions**					
19	I like to buy personalized drugs and tests on the internet					
20	I have access to my EHR (Electronic Health Record) and family medical history					
	**Financial Questions**					
21	With Personalized Medicine the amount of hospitalization days will be reduced					
22	Personalized drugs are more effective than standard drugs					
23	Side effects of personalized drugs are lower than that of standard drugs					
24	Personalized drugs are less expensive than standard drugs					
25	It is less expensive to develop personalized drugs than standard drugs					
26	Personalized Medicine can help society to save money					
27	Personalized Medicine should be covered by health insurance					
28	Medicare and Medicaid should cover the costs of Personalized Medicine					
29	The patient should pay out-of-pocket to cover the costs of Personalized Medicine					
	**Questions About Data Security**					
30	Exchange of biological samples and genetic data in the U.S. is secure					
31	Patients should have access to their genetic information					
32	Health insurer for life, disability and long-term care should be allowed to require genetic information					
33	Health insurance companies should have access to genetic information					
34	Employers should have access to genetic information of their employees					
35	I have a concern about the security of genetic data use					
36	I surf the internet every day					

Acceptable price difference between daily dose of standard and personalized drug is: $/%________

Acceptable price difference between treatment with standard and personalized therapy per day is: $/%________

Acceptable price for

Genetic test is: $____

Pharmacogenetic test is: $____

Protein test is: $____

Comment:

____

____

Additional Information:

Gender:	Male	☐	Female	☐		
Age:	20-30	☐	41-50	☐	61-70	☐
	31-40	☐	51-60	☐	over 70	☐
I am currently:	Student	☐	Employee	☐	Pensioner	☐
	University lecturer	☐	Non-employee	☐		
I am under medical treatment of:	cancer	☐	asthma	☐	other	☐
	diabetes	☐	depression	☐	none	☐
My healthcare expenses are covered by:	myself	☐	health insurance	☐		
	Medicare and Medicaid	☐	other	☐		
Of all my health expanses my health insurance covers:	30%	☐	80%	☐		
	50%	☐	100%	☐		
